# Donors in lung transplantation: does age matter?

**DOI:** 10.1093/icvts/ivad111

**Published:** 2023-07-08

**Authors:** Charlotte Ponte, Omar Alkhatiri, Anne Olland, Pierre-Emmanuel Falcoz

**Affiliations:** Hôpitaux Universitaire de Strasbourg, Service de chirurgie thoracique—Nouvel Hôpital Civil, Strasbourg, France; INSERM (French National Institute of Health and Medical Research), UMR 1260, Regenerative Nanomedicine (RNM), FMTS, Strasbourg, France; Université de Strasbourg, Faculté de médecine et pharmacie, Strasbourg, France; Hôpitaux Universitaire de Strasbourg, Service de chirurgie thoracique—Nouvel Hôpital Civil, Strasbourg, France; Université de Strasbourg, Faculté de médecine et pharmacie, Strasbourg, France; Hôpitaux Universitaire de Strasbourg, Service de chirurgie thoracique—Nouvel Hôpital Civil, Strasbourg, France; INSERM (French National Institute of Health and Medical Research), UMR 1260, Regenerative Nanomedicine (RNM), FMTS, Strasbourg, France; Université de Strasbourg, Faculté de médecine et pharmacie, Strasbourg, France; Hôpitaux Universitaire de Strasbourg, Service de chirurgie thoracique—Nouvel Hôpital Civil, Strasbourg, France; INSERM (French National Institute of Health and Medical Research), UMR 1260, Regenerative Nanomedicine (RNM), FMTS, Strasbourg, France; Université de Strasbourg, Faculté de médecine et pharmacie, Strasbourg, France

**Keywords:** Lung transplantation, Donors >60 years old, Outcomes, Respiratory function, Survival

## Abstract

A best-evidence topic was written according to a structured protocol. The question addressed was the following: in patient undergoing lung transplantation, are lungs from donors of age >60 years old (yo) associated with equivalent outcomes—including primary graft dysfunction, respiratory function and survival—than lungs from donors ≤60yo? Altogether, >200 papers were found using the reported search, of which 12 represented the best evidence to answer the clinical question. The authors, journals, dates, country of publication, patients group studied, study type, relevant outcomes, and results of these papers were tabulated. Amongst the 12 papers reviewed, survival results were different depending on whether donor age was analysed raw or adjusted for recipients’ age and initial diagnosis. Indeed, recipients with interstitial lung disease (ILD), pulmonary hypertension or cystic fibrosis (CF) had significantly inferior overall survival when receiving grafts from older donors. When older grafts are allocated to younger donors, a significant decrease in survival has been noticed in the case of single lung transplantation. In addition, 3 papers showed worse results regarding peak forced expiratory volume in 1 second (FEV1) in patients receiving older organs, and 4 showed comparable primary graft dysfunction incidence rates. We conclude that when carefully assessed and allocated to the recipient who could benefit most from the transplant (e.g., a patient with a diagnosis of chronic obstructive pulmonary disease (COPD), who would not require a prolonged cardiopulmonary bypass (CPB)), lung grafts from donors of >60yo offer comparable results to younger donors.

## INTRODUCTION

A best-evidence topic was constructed according to a structured protocol. This is fully described in the ICVTS [1].

## THREE-PART QUESTION

In [patients undergoing lung transplantation], are lungs from [donors > 60 years old] associated with [equivalent outcomes including primary graft dysfunction (PGD), respiratory function and survival] than lungs from donors ≤60 years old?

## CLINICAL SCENARIO

As a thoracic surgeon on call for lung procurement, you receive a call from the agency of health care. A graft from a 76-year-old (yo) male patient is offered. The cause of death is a sudden brain Haemorrhage. The clinical record shows no medical and no smoking history. The initial study shows a thoracic diameter similar to your patient’s one with a Pa02/Fi02 ratio evaluated at 420 mmHg. The CMV serology is negative. The CT-scan imaging shows no emphysema or suspicious nodules. The bronchoscopy shows perfect integrity of the airway. Then, you find a potential match for a 64yo man with COPD, but you hesitate to go through the lung transplantation (LTx) based on the donor’s age.

## SEARCH STRATEGY

Medline search using PubMed interface was performed with the following query: [lung transplantation OR lung transplant] AND [old donors OR age] AND [outcomes], with results only in English and including all types of publications. Afterwards, references from the retrieved studies were looked up manually.

## SEARCH OUTCOME

209 papers were found using the reported search. From these papers, 12 were selected, providing the best evidence to answer the question. These are presented in Table 1.

**Table 1:** Overview of the studies

Author, date, journal and country Study type (level of evidence)	Patient group	Outcomes	Key results	Comments/weaknesses
De Perrot *et al.*, 2007, J Thorac Cardiovasc Surg, Canada [2] Cohort study (level 3)	467 LTx Donors < 60yo: 407 Donors ≥60yo: 60 Period 1994–2005	30-Day mortality 10-Year survival	No significant difference (*P* = 0.09) Donors <60yo: 39% Donors ≥60yo: 16% (*P* = 0.07)	Retrospective single-centre study Small donors ≥ 60yo group size Initial experience with older lungs
Baldwin *et al.*, 2013, Am J Transplant, USA [3] Cohort study extracted from the OPTN database (level 3)	8860 LTx Donors <18yo: 937 Donors 18–30yo: 3218 Donors 30–54yo: 3837 Donors 55–64yo: 769 Donors ≥ 65yo: 99 Period 2005–2012	1-Year graft failure PGD	No significant difference No significant interaction of donor age (*P* > 0.50 in all groups)	Retrospective multicentre study No *P*-value concerning 1-year graft failure Recipients of lungs from donors ≥65yo excluded for secondary analysis
Shigemura *et al.*, 2014, Transplantation, USA [4] Cohort study (level 3)	593 LTx Donors <55yo: 506 Donors ≥55yo: 87 Period 2003–2009	30- and 90-day mortality, acute rejection in the 1st year Peak FEV1 (% predicted) Peak 6MWT Long-term survival	No significant differences Donors <55yo: 80.7 ± 28.8 Donors ≥55yo: 71.7 ± 37.1 (*P* < 0.05) Donors <55yo: 834 ± 286 Donors ≥55 years: 745 ± 197 (*P* < 0.05) No significant difference (*P* = 0.16)	Retrospective single-centre study Small size of the old donor group Different proportion of underlying lung disease between the groups
Sommer *et al.*, 2015, J Heart Lung Transplant, Germany [5] Cohort study (level 3)	440 bilateral LTx Donors <70yo: 413 Donors ≥70yo: 27 Period 2011–2016	1-Year FEV1 1-Year survival	Donors <70 years: 86.5 ± 26.2% Donors ≥70 years: 72.2 ± 23.8% (*P* = 0.01) No significant difference (*P* = 0.53)	Retrospective single-centre study Small number of patients in the subgroups Recipients of lungs from very old donors are older than recipients of lungs from donors <70yo (*P* < 0.0001) Short follow-up period
Hecker *et al.*, 2017, Eur J Cardiothorac Surg, Germany [6] Cohort study (level 3)	96 LTx Donor <60yo: 63 Donor 60–69yo: 17 Donor ≥70yo: 16 Period 2010–2016	Early outcomes (LOS, perioperative ECMO, PGD, mortality) 1-Year survival Best FEV1	No significant difference (No *P* available) Donors <60yo: 92% Donors 60–69 yo: 88% Donors ≥70yo: 93% No significant difference Highest for young donors (*P* < 0.005)	Retrospective single-centre study Small number of patients Narrow acceptance criteria Lungs from very old donors not transplanted to very young patients
Holley *et al.*, 2017, J Thorac Cardiovasc Surg, USA [7] Single institution cohort study (level 3)	882 adult LTx Overall cohort Donors <40yo: 555 Donors 40–54yo: 244 Donors ≥55yo: 83 LAS era (*n* = 396) Donors <40yo: 230 Donors 40–54yo: 108 Donors ≥55yo: 58 Period 1986–2016	1-Year survival 5-Year survival BOS-free survival Rejection-free survival PGD Grade III	Donors <40yo: 87% Donors 40–54yo: 87% Donors ≥55yo: 90% No significant difference Donors <40yo: 64% Donors 40–54yo: 61% Donors ≥55yo: 69% No significant difference No significant difference between age groups *P* = 0.131 No significant difference between age groups *P* = 0.953 Mean incidence: 16% No significant difference between age groups	Retrospective single-centre study Limited number of lungs from donors older than 60yo No data concerning BOS before LAS era
Schultz *et al.*, 2017, Transplant Proc, Denmark [8] Cohort study (level 3)	588 consecutive LTx (454 donors): Donors 0–39yo: 169 Donors 40–54yo: 203 Donors ≥55yo: 82 Period 1992–2012	Median survival (years) CLAD-free survival FEV1 (l) DLCO (mmol/min/kPa)	Donors 0–39yo: 8.14 Donors 40–54yo: 6.38 Donors ≥55yo: 3.33 (*P* = 0.001) Lowest with oldest donors *P* = 0.002 Donors 0–39yo: 2.89 Donors 40–54yo: 2.64 Donors ≥55yo: 2.32 (*P* = 0.01) Donors 0–39yo: 6.08 Donors 40–54yo: 5.64 Donors ≥55yo: 5.02 (*P* = 0.03)	Retrospective single-centre study Long experience in a single-centre study Heterogeneity of the data
Whited *et al.*, 2017, Ann Thorac Surg, USA [9] Cohort study extracted from the UNOS-STAR Database (level 3)	14,222 LTx Recipients ≤50yo: 3762 Donor <60yo: 3690 Donor >60yo: 72 Recipients >50yo: 10 460 Donor <60yo: 9969 Donor >60yo: 491 Period 2005–2014	1-Year survival 5-Year survival PGD	Donors >60yo: 78% Donors <60yo: 83% (*P* < 0.001) Donors >60yo: 44% Donors <60yo: 52% (*P* < 0.001) After stratification by recipient age, *P* = 0.123 No significant difference between age groups	Retrospective multicentre study Only 2% of the younger cohort received lungs from donors >60yo and 5% of the older cohort did Stratification by age and by type of transplant (simple vs double)
Hall *et al.*, 2019, Ann Thorac Surg, USA [10] Cohort study extracted from the UNOS-STAR Database (level 3)	15 844 LTx: Donors < 18yo: 1477 Donors 18–29yo: 5743 Donors 30–39yo: 2710 Donors 40–49yo: 2938 Donors 50–59yo: 2326 Donors >60yo: 650 Period 2005–2015	OS Incidence of rejection	Donors >60yo had the worst survival (*P* < 0.0001) No difference in survival based on the combination of donor and recipient ages No significant correlation (*P* = 0.228)	Retrospective multicentre study No data concerning short-term impacts Case-by-case analysis of donor factors
Auråen *et al.*, 2019, Transplantation, Denmark, Finland, Norway, Sweden [11] Cohort study (level 3)	913 primary double LTx Donors <55yo: 657 Donors ≥55yo: 256 Period 2000–2013	ICU LOS 90-Day survival 1-Year survival OS	Significantly higher when donor lungs ≥55yo (*P* = 0.018) *P* = 0.139 *P* = 0.451 *P* = 0.278 No significant difference between age groups	Retrospective multicentre study Association between donor age and survival differed significantly by recipient diagnosis Only few recipients older than 65 years old No data concerning PGD, time on ventilator, spirometry, acute rejection
Renard *et al.*, 2020, Ann Thorac Surg, France [12] Cohort study (level 3)	241 LTx: Donors <65yo: 197 Donors ≥65yo: 44 Period 2014–2019	1-Year survival 5-Year survival PGD Grade III 1-Year FEV1 5-Year CLAD-free survival	Donors <65yo: 77% Donors ≥65yo: 67% (*P* = 0.41) Donors <65yo: 49% Donors ≥65yo: 50% (*P* = 0.41) Non-significant difference (*P* = 0.99) Non-significant difference (*P* = 0.68) Donors <65yo: 26% Donors ≥65yo: 31% (*P* = 0.99)	Retrospective single-centre study Restrictive donor acceptance policy: less smoking history, less bronchoscopic abnormality, less chest opacity Short follow-up
Vanluyten *et al.*, 2023, Ann Surg, Belgium [13] Single institution retrospective study (level 3)	647 bilateral LTx: Donors ≥70yo: 69 Propensity matched younger donor (<70yo) cohort: 69	ICU LOS PGD Grade III 5-Year survival 5-Year CLAD-free survival	Donors <70yo: 6 days Donors ≥70yo: 7 days (*P* = 0.46) Donors <70yo: 29% Donors ≥70yo: 26% (*P* = 0.85) Donors <70yo: 73.1% Donors ≥70yo: 73.6% (*P* = 0.72) Donors <70yo: 59.2% Donors ≥70yo: 51.5% (*P* = 0.41)	Retrospective single-centre study Restrictive donor acceptance policy Small number of patients

BOS: bronchiolitis obliterans syndrome; CLAD: chronic lung allograft dysfunction; ECMO: extracorporeal membrane of oxygenation; FEV1: forced expiratory volume in 1 s; ICU: intensive care unit; LAS: lung allocation score; LOS: length of stay; LTx: lung transplantation; OPTN: Organ Procurement and Transplantation Network; OS: overall survival; PGD: primary graft dysfunction; STAR: standard transplant analysis and research; UNOS: United Network of Organ Sharing; yo: years old; 6MWT: 6-min walking test.

## RESULTS

De Perrot *et al.* [2] analysed a 467 patients’ cohort divided into recipients of lungs from donors ≥60yo and recipients of lungs from donors <60yo. Although 30-day mortality and 10-year survival tended to be worse in recipients of older lungs, it did not reach statistical significance. Interestingly, 30-day mortality tended to be higher in recipients of older lungs presenting with pulmonary fibrosis and in pulmonary hypertension patients. The authors concluded that lungs from older donors could be considered for LTx if they fulfil all other lung donor selection criteria.

Baldwin *et al.* [3] evaluated an 8,860 patients cohort to study the impact of donor age on 1-year graft survival and the incidence of grade III PGD. The difference in 1-year graft failure was not statistically significant among recipients of lungs from donors aged 55–64 years except for those severely ill or receiving preoperative mechanical ventilation. Lungs from donors aged ≥65yo showed an increased rate of 1-year graft failure. It was, however, a small subgroup as it only included 99 patients. The authors did not find an association between donor age and the risk of PGD. They concluded that there was a continuous relationship between donor age and the relative hazard of graft failure in the first year after LTx.

Shigemura *et al.* [4] published the results of a 593 patients cohort divided into 2 groups of donors: 506 donors <55yo and 87 donors ≥55yo. Allograft quality was evaluated by measuring peak FEV1 and a 6-min walk test (6MWT). Although early measurements were significantly worse for older grafts, authors only showed a significant reduction of peak FEV1 in older lung recipients. The authors showed similar 3- and 5-year survival for both groups. Multivariate analysis of the recipients of lungs from older donors demonstrated that previous pulmonary hypertension and prolonged intraoperative CPB time were significant risk factors for mortality. The authors demonstrated that transplanting lungs from donors >55yo did not yield worse short or long-term outcomes as compared with transplanting lungs from younger donors.

Sommer *et al.* [5] studied the postoperative FEV1, 6MWT, and survival of a 440 patients cohort. They only considered double LTx. Older donors were pre-selected for various criteria: less often positive for smoking history or abnormal bronchoscopy results. Results showed a similar time of mechanical ventilation, length of stay (LOS) in the intensive care unit (ICU), total LOS and PGD rates. Recipients of donor's lungs <70yo performed better at the 6MWT. When accounting for underlying diagnosis, emphysema patients performed similarly, whereas patients affected with interstitial pulmonary fibrosis receiving older lungs achieved significantly shorter distances and lower FEV1. The authors concluded that lung grafts from selected donors aged > 70yo can safely be used for LTx without impairing the recipients’ postoperative morbidity or mortality. Recipients with the underlying diagnosis of emphysema benefitted better than other recipients from transplantation with older lungs.

In 2017, Hecker *et al.* [6] investigated the impact of using lungs from very old donors aged >70 years on early and late outcomes after LTx. They demonstrated that the use of old (60–69yo) and very old (≥70yo) lungs did not significantly affect recipient’s survival. PGD rates at 72 h remained comparable but treated acute rejection was highest for older organs (*P* < 0.05). Regarding long-term outcomes, recipients of young, old and very old lungs showed similar 1- and 3-year survival rates.

Holley *et al.* [7] published a retrospective single-institution cohort study. They studied 882 adults who underwent LTx from 1986 to 2016. Unadjusted survival did not differ significantly between donor age categories. Proportional hazards analysis of donor age showed no association between donor age and recipient survival or bronchiolitis obliterans syndrome-free survival. Moreover, adjusted proportional hazards analysis showed that donor age was not associated with impaired 10-year survival. Nevertheless, donors >60yo did exhibit a trend towards greater ventilator, ICU, and total LOS. This finding suggested that although the older donor lungs did not result in differences in survival or early graft function, they could contribute to increased resource use after the transplant.

Schultz *et al.* [8] published their single-centre retrospective study about the impact of age and donor smoking history on allograft function, on chronic lung allograft disease (CLAD)-free survival and overall long-term survival. 454 donors were divided in 3 groups: <40yo donors, donors aged from 40 to 54yo and donors ≥55yo. The baseline values of FEV1 and DLCO were significantly higher in recipients receiving lungs from donors <40yo. Median survival decreased in a significant way when donors get older. Similarly, authors found statistically significant inferior CLAD-free survival when receiving organs from donors >55yo. In a subgroup analysis of the oldest donors, there was no difference in survival and CLAD-free survival when comparing donors 55 to 60yo to donors >60yo.

Whited *et al.* [9] aimed to examine the impact of transplanting lungs from donors > 60yo in post-transplant survival in a cohort of 14 222 patients. The cohort was divided into 2 groups: recipients aged <50yo and recipients aged 50yo and more. The overall survival (OS) was worse among patients receiving lungs from donors >60yo. When the younger recipients were sub-stratified by double versus single LTX, the use of older donors was associated with a significant decrease in survival only in case of single LTx. This effect was not as pronounced in the older recipient cohort, as there was a small but statistically significant decrease in survival in both the double and single LTx groups. The authors demonstrated acceptable outcomes using older donor lungs in younger patients if double LTx was performed.

In 2019, Hall *et al.* [10] retrospectively studied a cohort of 15 844 patients who underwent LTx from 2005 to 2015. Donors >60yo had the worst survival of all donor age ranges. When analysing survival based on the combination of donor and recipient ages, there was no difference in OS except for recipients aged 60–69. Propensity score matching was performed by donors <60yo and ≥60yo and did not show any significant difference in survival. Considering the incidence of acute rejection, no correlation with donor age was observed. Authors concluded that donor age alone should not be a reason for declining lungs, even when considering older recipient age ranges and that the combination of donor and recipient age is important in LTx.

Auråen *et al.* [11] published their multicentric retrospective cohort study about outcomes of LTx from old donors. They reviewed all their bilateral LTx from donors ≥55yo, stratified by recipient primary diagnosis, to compare outcomes with transplantations from younger donors. They showed no difference in OS among patients transplanted from donors ≥55yo compared with younger donors. However, in cystic fibrosis recipients, donor ≥55yo was associated with inferior 1-year and OS. Recipients with interstitial lung disease receiving organs from donors ≥ 55yo had significantly inferior 90-day, 1-year survival, and OS compared with those receiving organs from younger donors. Utilization of older donors was associated with increased ICU LOS for recipients with CF and ILD, but not in the other groups.

Renard *et al.* [12] studied a cohort of 241 patients who underwent LTx. 44 patients received grafts from donors ≥65yo (elderly group) and were compared to those from donors <65yo (control group). The authors performed a more selective policy regarding organ quality when accepting lungs from old donors. They did not find any significant difference between groups in any outcomes studied, including PGD, 30-day mortality, 1-year survival, CLAD-free survival and 5-year OS. Regarding allograft function, the FEV1 did not differ significantly between elderly and control groups at 6 months and 1 year. The authors concluded that carefully selected lung grafts from donors ≥65yo are associated with similar early outcomes than grafts from younger donors.

Vanluyten *et al.* [13] published their single-centre retrospective analysis concerning outcomes of LTx from donors ≥70yo. Out of 647 bilateral LTx, 69 were performed from donors ≥70yo. They aimed to compare short- and long-term outcomes with a propensity-matched younger donor (<70yo) cohort. The authors performed a selective policy regarding organ quality when accepting lungs from old donors and were more inclined to assign lungs from elderly donors to stable older patients with COPD. Postoperative course was similar for both groups and no statistically significant differences were observed concerning ventilation, ICU LOS and PGD. Furthermore, they observed no significant difference in spirometry results, in 5-year and CLAD-free survival. Authors concluded that accepting grafts from selected donors >70yo can be safe and feasible but donor age should be perceived as a risk factor that needs to be balanced against the urgency for transplantation.

## CLINICAL BOTTOM LINE

We are here chronologically presenting data from cohorts studied between 1986 and 2019. The patients with the oldest data are included in large cohorts and the authors of the studies captured sufficient data for a robust statistical analysis. Considering the level of evidence, all the studies are retrospective non-randomized studies. Nevertheless, we looked for large cohort studies that analysed donor age as a binary variable. The data presented here are showing that when analysed as a binary variable and without adjusting for the presence of other extended donor criteria, old donor age can be associated with worse OS. However, when the recipient's age and underlying diagnosis were considered, these results almost consistently lost their statistical significance. Some authors have sub-stratified patients by single or bilateral LTx. In this way, in younger recipients, the use of older donors was associated with a similar survival when performing double LTx and a significant decrease in survival performing single LTx. Nevertheless, it has been noticed that double lung transplant was the dominant procedure in the younger recipients’ cohorts and the single transplant was the dominant procedure in the older recipients’ cohorts. Regarding respiratory functions, results are showing better short- and long-term postoperative peak FEV1 from younger lungs. The donor age was not associated with PGD risk in the papers presented. Considering CLAD, there are data available. However, some authors described a significant difference in CLAD-free survival when using older donors.

Hence, during the allocation process, lungs from donors ≥60yo should not be systematically rejected based on this parameter alone and the recipients’ characteristics should also be considered. From the data collected here, the proper recipients for lungs from old donors appear to be emphysema patients with no pulmonary hypertension and >60 years old. Thus, accepting lung grafts from older donors can be a safe and feasible strategy but donor age should be perceived as a risk factor that needs to be balanced against the urgency for transplantation.
